# Pulp in Shop-Bought Orange Juice Has Little Effect on Flavonoid Content and Gut Bacterial Flavanone Degradation *In Vitro*

**DOI:** 10.1007/s11130-019-00739-5

**Published:** 2019-06-21

**Authors:** Min Hou, Emilie Combet, Christine Ann Edwards

**Affiliations:** 1 Human Nutrition, School of Medicine, Dentistry and Nursing, College of Medical, Veterinary & Life Sciences University of Glasgow, New Lister Building, Glasgow Royal Infirmary, 10-16 Alexandra Parade, Glasgow, G31 2ER Scotland, UK; 2 School of Public Health, College of Medicine, Shanghai Jiao Tong University, Shanghai, China

**Keywords:** Colonic fermentation, Gastrointestinal digestion, Orange juice flavanones, Phenolic catabolites, Pulp

## Abstract

Orange juice is an important source of flavanones in the Western diet. However, little is known of the variation in flavanone content of shop-bought orange juice with pulp (OJP) or without pulp (OJ), nor the impact of pulp on the fate of flavanones in the gut. Total phenols, total flavonoids, antioxidant capacity, hesperidin and narirutin, and dietary fibre were measured in six orange juice brands sold as OJP and OJ. The inclusion of pulp had little impact on fibre content. Apart from total phenols (OJ: 208.4 ± 10.7 μg gallic acid equivalents (GAE) ml^−1^; OJP: 225.9 ± 16.7 μg GAE ml^−1^, *P* < 0.05), there were no differences between OJ and OJP. The fate of flavanones in OJ and OJP (Tropicana) were further compared using *in vitro* gastrointestinal (GI) models. After *in vitro* upper GI digestion, recovery of hesperidin was higher in OJ compared with OJP (89 ± 6 vs. 68 ± 3%, *P* = 0.033). After 2 h colonic fermentation, hesperidin was 1.2 fold higher in OJP than OJ. However, after 24 h colonic fermentation there was no significant difference between juices in terms of hesperidin, hesperetin, narirutin, naringenin and catabolites. In conclusion, the amount of pulp included in these shop-bought orange juices had little impact on flavanone metabolism in models of the GI tract. The effects of greater amounts of orange pulp remain to be determined.

**Electronic supplementary material:**

The online version of this article (10.1007/s11130-019-00739-5) contains supplementary material, which is available to authorized users.

## Introduction

Orange juice is widely consumed and has been associated with a range of potential health effects including anti-inflammatory actions [1, 2], positive impact on cardiovascular risk factors [3–5], and cognitive function [6]. Orange juice is a rich source of polyphenols mainly flavanones (hesperidin and narirutin) which, along with their catabolites, may contribute substantially to the health effects of the juice. To elucidate dietary intakes for epidemiological studies and to develop nutritional advice, it is necessary to understand 1) how flavanone content varies between sources, and 2) how different formulations affect flavanone metabolism and catabolite release in the GI tract.

According to the Phenol-Explorer database [7], the content of hesperidin (4-73 mg/100 ml) and narirutin (2–15 mg/100 ml) in pure orange juice (blond) varies substantially. The bioavailability of flavanones also varies considerably with 0.8–16% of ingested dose excreted in urine depending on the OJ tested [8–14]. This means that most of the ingested flavanones reach the colon. In juice, flavanones are present mainly as glycosides [4], and can be absorbed into circulatory system only after deglycosylation and degradation by intestinal enzymes or bacteria [11, 15–17]. In the colon, the aglycones (hesperetin, naringenin) are released by bacterial *β*-glucosidase, *α*-rhamnosidase and *β*-glucuronidase [18]. Clostridia, eubacteria, bifidobacteria and lactobacilli can then use ring-cleavage, decarboxylation, demethylation, and dehydroxylation [18–21] to transform flavonoids into bioavailable and bioactive phenolic acids [17].

Orange juice is now commonly marketed as smooth (no pulp) juice and juice with pulp (or ‘with bits’ in the UK) (OJP). The pulp is derived from the cellular tissues of the endocarp [22]. There has been suggestion from studies using juices with extra added pulp that this adds substantially to flavanone content [23, 24]. Polyphenols closely physically associated with dietary fibre are at least partially bioavailable [25]. However, it is not clear if shop bought juices with pulp (OJP) have the same impact nor if the addition of pulp in these juices impacts on the bioaccessibility, metabolism and catabolism of the flavanones in the small and large intestine. In this study, the stability, release and degradation of flavanones were assessed in UK shop-bought juices formulated with and without pulp using *in vitro* models of GI digestion and colonic fermentation.

## Materials and Methods

### Chemicals

Full details of chemicals and standards used are in online supplementary files.

### Orange Juices for Analysis

A selection six brands of OJ and OJP (Table 1; labelled 100% freshly squeezed, not from concentrate) were purchased from local supermarkets in Glasgow, UK between April of 2013 and March of 2014. Additional details are in the supplementary file.

**Table 1:** Total phenolic content (μg GAE ml^−1^), total flavonoids (μg QE ml^−1^), antioxidant capacity (mM Fe^2+^), narirutin (mg/100 ml), hesperidin (mg/100 ml) and dietary fibre content (g/100 ml) in orange juices

Brand	Type	Total phenols	Total flavonoids	FRAP	Narirutin	Hesperidin	Dietary fibre
Tropicana	OJ	216.5 ± 12.0	76.8 ± 0.5	5.9 ± 0.3	4.2 ± 0.2	37.3 ± 1.8	0.1
	OJP	228.4 ± 5.9	86.7 ± 17.1	6.4 ± 0.4	4.9 ± 0.3	40.5 ± 1.6	0.1
Sainsbury’s	OJ	213.5 ± 2.7	84.9 ± 13.6	6.0 ± 0.1	2.3 ± 0.1	34.5 ± 0.5	0.1
	OJP	252.3 ± 6.6	100.2 ± 15.0	7.4 ± 0.4	3.7 ± 0.3	40 ± 1.5	0.3
Tesco	OJ	220.9 ± 1.9	79.2 ± 3.3	6.3 ± 0.5	3.7 ± 0.1	38 ± 0.8	0.1
	OJP	226.1 ± 5.9	78.9 ± 7.7	6.1 ± 0.6	4.1 ± 0.1	41.9 ± 1.5	0.2
Innocent	OJ	197.2 ± 9.1	82.5 ± 5.2	4.6 ± 0.3	4.4 ± 0.2	30 ± 1.2	0.1
	OJP	199.8 ± 9.6	81.9 ± 3.5	5.0 ± 0.2	5.3 ± 0.1	37.4 ± 1.9	0.2
Morrison’s	OJ	207.8 ± 3.2	86.4 ± 1.2	5.2 ± 0.1	7.8 ± 0.1	21.5 ± 1.0	0.1
	OJP	224.0 ± 7.7	101.1 ± 7.0	5.3 ± 0.1	9.6 ± 0.9	21 ± 1.7	0.2
Waitrose	OJ	194.5 ± 8.2	78.6 ± 12.9	4.9 ± 0.3	2.6 ± 0.1	17.8 ± 0.2	0.3
	OJP	224.6 ± 9.1	108.9 ± 14.6	5.2 ± 0.1	2.8 ± 0.4	18.5 ± 1.3	0.2
Mean	OJ	208.4 ± 10.7	81.4 ± 3.8	5.5 ± 0.7	3.9 ± 1.4	20.0 ± 9.1	0.1 ± 0.1
	OJP	225.9 ± 16.7 ^a^	93.0 ± 12.1	5.9 ± 0.9	5.1 ± 2.4	32.9 ± 10.3	0.2 ± 0.1

Data expressed as mean of triplicate values ± SEM. Paired t-test was performed to determine difference between OJ and OJP

*OJ* orange juice without pulp; *OJP* orange juice with pulp

^a^ Significantly different from OJ, *p* < 0.05

### Analysis of Fibre in OJ and OJP

The fibre content of each orange juice was analysed by using AOAC method [26] by the commercial food testing facility at the School of Health and Life Science, Glasgow Caledonian University, Glasgow, UK.

### Analysis of Total Flavonoids, Total Phenols, Antioxidant Capacity

Total flavonoids were determined in orange juices by spectrophotometric method [27]. Total phenols in orange juices and samples from *in vitro* GI digestion was determined by the Folin-Ciocalteau method [28]. Antioxidant capacity was determined in orange juices and samples from *in vitro* GI digestion using the FRAP assay [29]. See supplementary material for additional details.

### *In Vitro* Gastrointestinal Digestion Model

Tropicana juices (OJ and OJP) were selected as a representative and popular brand in the UK for investigating *in vitro* digestion and fermentation of orange juice in the gut models. The volume of orange juice used in the models was proportional to the ingestion of 250 ml juice [30]. The model of the upper GI digestion was adapted from Gil-Izquierdo [[31]; supplementary file]. This model has many similarities to the INFOGEST model [32] but there are some variations. There was no saliva phase as the test food is a liquid, and there was no phospholipid in the gastric phase. The gastric phase ran for 1 h, not 2 h as for the INFOGEST model, and at pH 2 rather than pH 3. The small intestinal phase was incubated for 4 h, not 2 h. This reflects the liquid nature of our test meal and time required for arrival of the food in the large intestine, which is mimicked by the fermentation model (phase three in our study). Pancreatin and bile extract were included, as suggested by INFOGEST (see supplementary material for additional details). The GI digestion was conducted in triplicate and after digestion the resultant fluid was dialysed (MW cut off 0.1–0.5 kDa, Sigma Poole, UK) to remove sugars, dextrins, fatty acids and peptides normally absorbed in the small intestine. Flavonoids would remain (Hesperidin MW 610, Narirutin MW 580). Dialysed samples were freeze-dried (Edwards Micro Modulyo Crawley UK) before transferring to the fermentation model. Samples for the evaluation of stability / release of hesperidin and narirutin from orange juice during digestion were not dialysed.

### *In Vitro* Fermentation Model with Human Colonic Bacteria

Faecal samples were obtained from six healthy Caucasian volunteers (four women and two men, 23 ± 2.6 years, 67 ± 12.5 kg, BMI 22.5 ± 2.3) who were non-smokers, had a normal diet, no digestive diseases, no food allergies, and did not receive antibiotics for six months prior to recruitment. Volunteers followed a low-polyphenol diet for two days, avoiding fruits, vegetables, tea, coffee, wine and high fibre foods before providing a faecal sample. The model of colonic fermentation was adapted from Jaganath et al. [33].

### Analysis of Hesperidin, Hesperetin, Narirutin and Naringenin in Orange Juice, Digesta and Fermented Samples

#### Extraction of OJ and OJP

A liquid-liquid method was used to extract hesperidin and narirutin from orange juice (see supplementary material for additional details).

#### Extraction of the Gastric Digesta and the Fermented Samples

A liquid-liquid method was used to extract hesperidin and narirutin from the gastric digesta, and hesperidin, narirutin, hesperetin and naringenin from the fermented samples (see supplementary material for additional details).

### Analysis of Hesperidin, Hesperetin, Narirutin and Naringenin by HPLC-PDA

Analysis of hesperidin, hesperetin, narirutin and naringenin was performed as described by Pereira-Caro et al. [11] (see supplementary material for additional details).

### Extraction and Derivatisation of Phenolic Acids in Fermented Samples

Phenolic acid extraction, derivatisation and analysis were performed as described by Combet et al. [34] (see supplementary material for additional details).

### Analysis of Phenolic Acids in Fermented Samples by GC-MS

To analyse phenolic acids in fermented samples, we used a GC-MS method (see supplementary material for additional details).

### Statistical Analyses

All statistical analyses were performed using SPSS statistics software (SPSS version 22, IBM Corporation, Somers, USA). Data were assessed for normality of distribution using the Anderson-Darling test. Data were expressed as mean values ± SEM. Paired *t*-tests or repeated measures ANOVA with Tukey *post hoc* tests were used to determine significant differences as *p* < 0.05. The *in vitro* gastrointestinal digestion phase was conducted as 2 × 3 and the fermentation with human colonic bacteria was conducted as 2 × 6.

## Results and Discussion

### Flavanone Content of Orange Juices

The total content of hesperidin and narirutin in six brands of shop-bought orange juice ranged from 21.3 to 46.0 mg/100 ml and there was no significant difference in hesperidin and narirutin between OJ and OJP (20 ± 9.1 mg/100 ml vs. 32.9 ± 10.3 mg/100 ml; 3.9 ± 1.4 mg/100 ml vs. 5.1 ± 2.4 mg/100 ml). Overall, the OJP had higher total phenolic compounds than OJ (225.9 ± 7.5 μg GAE ml^−1^ vs. 208.4 ± 6.1 μg GAE ml^−1^) (Table 1). This may be due to non-flavanone compounds such as flavanols, flavones and phenolic acids [34]. There was no difference between OJ and OJP for all other variables. The fibre content ranged from 0.1 to 0.3 g 100 ml, and was similar between smooth OJ and OJP (Table 1). The fibre content of each juice was measured only once, but the low values obtained were similar between brands and with the declared fibre content by the manufacturers. The pulp has been suggested to be rich in flavonoids [3]; however, in this study, there was little impact of the inclusion of pulp on the extractable flavanone content in these shop-bought juices. Pellet weight after centrifugation of the OJP juices in this study differed by 2–3% total juice compared to OJ. This is in contrast to the 33% content for freshly squeezed oranges. In Rangel-Huerta’s study [3], 5.5 g of pulp containing 4.5 g fibre was added to 500 ml orange juice and more than doubled the amount of flavanones over their standard juice. This is 20 times the dietary fibre in the shop-bought juices used in this study. Thus the lack of effect of OJP on flavanone content may be due to the low levels of pulp in these shop-bought juices. The amount of pulp added is not declared.

### Impact of *In Vitro* GI Digestion on Orange Juice Hesperidin and Narirutin

In the gastric phase model, hesperidin was 1.3 and 1.2 fold higher in the simulated gastric fluid (at beginning and after 1 h) with OJP than with OJ with no change in the narirutin content (Table 2). After small intestinal digestion, the recovery of hesperidin was significantly higher in OJ than OJP (89 ± 6% and 68 ± 3% of original amount) and the narirutin recovery was 55 ± 1% and 57 ± 4% respectively (Table 2).

Tagliazucchi et al. reported that polyphenols (mainly anthocyanins) in grapes decreased by 44% when transiting from an acidic gastric model to the alkaline small intestinal *in vitro* digestion model [35]. Another study reported that gastric digestion reduced naringenin, caffeic acid, ferulic acid, *p*-coumaric acid, and 4-hydroxybenzoic acid by 5–36%, but increased chlorogenic acid, sinapic acid, rutin and (+)-catechin by 5–58%, rather than hesperidin and quercetin in a blended fruit juice containing orange, kiwi, pineapple and mango [36]. In our study, hesperidin was higher in the gastric digesta of OJ compared to OJP, and less hesperidin was recovered from the small intestine model digesta with OJP compared with OJ (even though hesperidin content was similar in the two juice types). This suggests that hesperidin from orange juice with pulp was not as stable in the gastric phase.

Previous *in vitro* studies have found transformation of orange juice flavanone into chalcone under gastrointestinal conditions [31]. Although hesperidin content was not significantly different between orange juices, after upper GI digestion, recovery of hesperidin was higher in OJ than OJP (Table 2), which may be related to transformation or precipitation of hesperidin. In addition, pulp affected the reduction in hesperidin when transferring from the gastric to alkaline intestinal model in the present study.

### Changes in Total Phenolic Content and Antioxidant Capacity during GI Digestion *In Vitro*

At 0 h in the upper GI digestion model, OJ had 1.6 fold higher antioxidant capacity compared with OJP despite a lower hesperidin content. The antioxidant capacity in orange juice is not dependent just on hesperidin or flavanones and there may be food matrix effects [37]. After the upper GI digestion, the total phenolic content was17.2 ± 7.2 mg GAE from 40 ml of OJ and 18.5 ± 1.6 mg GAE from 40 ml of OJP (Table 2).

**Table 2:** Hesperidin and narirution content / discovery (mg from 40 ml of orange juice), total phenol (mg GAE from 40 ml of orange juice) and antioxidant capacity (mM Fe ^2+^) in gastric digesta during the upper gastrointestinal digestion

Gastric digestion												
	0 h				1 h							
	Hesperidin	Narirutin	Total phenol	FRAP	Hesperidin	Narirutin	Total phenol	FRAP				
OJ	4.2 ± 0.4	0.3 ± 0.0	24.0 ± 3.2	1.1 ± 0.0	4.5 ± 0.1	0.3 ± 0.0	28.3 ± 0.3	1.0 ± 0.0				
Recovery (%)	N/A	N/A	N/A	N/A	109.2 ± 8.5	97.0 ± 7.0	N/A	N/A				
OJP	5.3 ± 0.1 ^a^	0.3 ± 0.0	27.6 ± 2.3	0.7 ± 0.0 ^a^	5.4 ± 0.2 ^a^	0.3 ± 0.0	31.4 ± 1.2	0.7 ± 0.0 ^a^				
Recovery (%)	N/A	N/A	N/A	N/A	102.4 ± 3.4	96.4 ± 2.1	N/A	N/A				
Small intestinal digestion												
	2 h				4 h				6 h			
	Hesperidin	Narirutin	Total phenol	FRAP	Hesperidin	Narirutin	Total phenol	FRAP	Hesperidin	Narirutin	Total phenol	FRAP
OJ	3.3 ± 0.3	0.2 ± 0.0	17.9 ± 0.4	0.2 ± 0.0	3.6 ± 0.2	0.2 ± 0.0	19.6 ± 3.0	0.2 ± 0.0	3.7 ± 0.1	0.2 ± 0.0	17.2 ± 1.2	0.2 ± 0.0
Recovery (%)	N/A	N/A	N/A	N/A	N/A	N/A	N/A	N/A	89.0 ± 5.8	54.8 ± 0.9		
OJP	3.5 ± 0.1	0.2 ± 0.0	23.9 ± 0.5	0.2 ± 0.0	4.0 ± 0.2 ^a^	0.2 ± 0.0	23.6 ± 0.3	0.2 ± 0.0	3.6 ± 0.2	0.2 ± 0.0	18.5 ± 1.6	0.2 ± 0.0
Recovery (%)	N/A	N/A	N/A	N/A	N/A	N/A	N/A	N/A	67.9 ± 3.2	56.5 ± 3.7		

Values are expressed as mean of three incubations ± SEM (*n* = 3), Repeated measures ANOVA with Tukey *post hoc* was performed to determine significant difference

*OJ* orange juice without pulp; *OJP* orange juice with pulp

^a^Significantly different from OJ, *p* < 0.05; N/A: not applicable

Cilla et al. [38] reported that the total phenolic content of fruit beverages was reduced by 47% during *in vitro* GI digestion, which is consistent with our findings where we saw a reduction of total phenolic content of 31 and 36%, respectively in OJ and OJP after the upper GI digestion. The pulp could contain small amounts of protein, fat and ash [39], and connective tissues which may influence the phenolic contents during the GI digestion. The effects of these matrices during the GI digestion should be investigated further.

Furthermore, the higher total phenol content in OJP might indicate that addition of pulp increases release of total phenols under upper GI conditions, even though the pulp does not contain much fibre. In addition, the antioxidant capacity decreased respectively by 56 and 42% in OJ and OJP. Alexandropoulou et al. [40] investigated effects of protein and iron on the antioxidant capacity of green tea under conditions of digestion *in vitro*. As in the present study, the reduction of antioxidant capacity may be due to the interactions between antioxidants and compounds in the upper GI digestion system, but was not affected by the pulp. The mechanism should be further studied.

### Degradation of Hesperidin and Narirutin during Colonic Fermentation *In Vitro*

After 24 h fermentation, total phenolic contents of the fermentation fluid was similar between OJ and OJP (4.5 ± 0.8 vs. 4.9 ± 1.0 mM GAE) (Table 3). Percentages of degradation of hesperidin and narirutin were similar after 24 h fermentation by gut bacteria. There was no difference in production of hesperetin and naringenin from OJ and OJP (Table 3). This suggests that the pulp had no impact on degradation of orange juice flavanone by gut bacteria. Hesperidin was marginally higher by 14.9 μM in the incubation with digested OJP compared with OJ at 2 h (Table 3). It may be due to increased release of hesperidin from the pulp under upper GI conditions or complexes of flavanone binding to pulp under GI conditions.

**Table 3:** Narirutin, hesperidin, naringenin and hesperetin (μM), total phenols (mM GAE) in cultures of orange juices during *in vitro* fermentation with human faecal bacteria

		Narirutin	Hesperidin	Naringenin	Hesperetin	Total phenol
0 h	OJ	2.7 ± 0.6	68.4 ± 14.1	1.1 ± 0.4	0.7 ± 0.3	5.3 ± 0.2
	OJP	3.8 ± 0.6	103.1 ± 9.8	1.5 ± 0.5	0.9 ± 0.1	4.6 ± 0.7
2 h	OJ	2.5 ± 0.7	75.5 ± 9.0	3.0 ± 0.5	2.1 ± 0.5	5.1 ± 0.4
	OJP	2.8 ± 0.6	90.4 ± 9.5 ^*^	2.7 ± 0.8	2.3 ± 0.3	5.6 ± 0.2
4 h	OJ	2.4 ± 0.7	73.9 ± 8.6	3.3 ± 0.9	1.9 ± 0.9	4.9 ± 0.7
	OJP	2.7 ± 0.6	84.9 ± 12.7	4.1 ± 1.0	4.5 ± 0.4	5.0 ± 0.8
6 h	OJ	2.3 ± 0.7	68.9 ± 13.0	3.8 ± 1.1	3.6 ± 1.1	5.2 ± 0.4
	OJP	2.0 ± 0.5	78.4 ± 14.8	3.1 ± 0.7	3.6 ± 0.4	5.5 ± 0.4
24 h	OJ	1.4 ± 0.5	42.9 ± 12.4	3.7 ± 0.9	4.6 ± 1.9	4.5 ± 0.8
	OJP	1.4 ± 0.4	51.7 ± 19.3	3.6 ± 1.0	5.1 ± 1.8	4.9 ± 1.0

Data expressed as mean of six fermentations ± SEM (*n* = 6), Repeated measures ANOVA with Tukey *post*-*hoc* was performed

*OJ* orange juice without pulp; *OJP* orange juice with pulp

^a^ Significantly different from OJ, *p* < 0.05

### Production of Phenolic Acids during Colonic Fermentation *In Vitro*

**Fig. 1:**
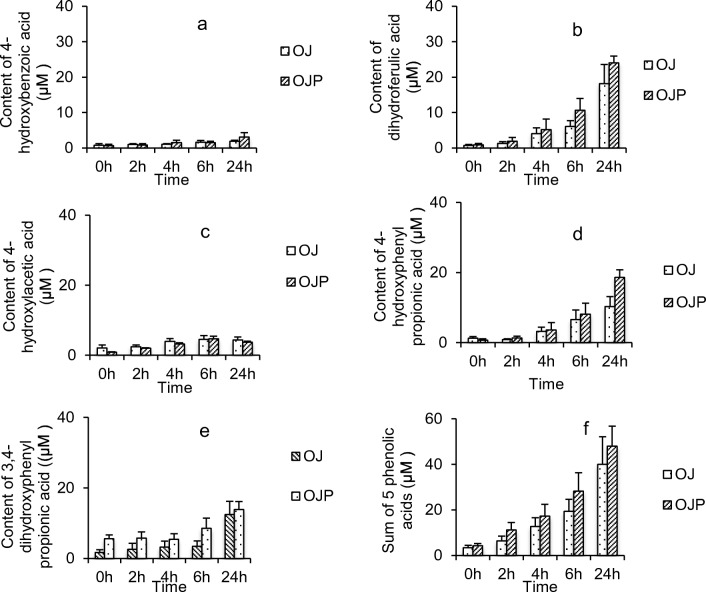
Contents of 4-hydroxybenzoic acid (**a**), dihydroferulic acid (**b**), 4-hydroxyphenylacetic acid (**c**), 4-hydrophenylpropionic acid (**d**), 3,4-dihydroxyphenylpropionic acid (**e**) and sum of these five phenolic acids (**f**) (μM) during 24 h fermentation with gut bacteria. Data expressed as mean values ± SEM (*n* = 6)

Five phenolic acids (4-hydroxybenzoic acid, 4-hydroxyphenylacetic acid, 4-hydrophenylpropionic acid, dihydroferulic acid and 3,4-dihydroxyphenylpropionic acid) was derived from metabolism of orange juices by gut bacteria (Fig. 1). The total amount of five phenolic acids was similar between OJ and OJP during simulated colonic fermentation (40.0 ± 11.4 μM and 48.0 ± 8.9 μM) (Fig. 1). This indicated that adding pulp did not influence degradation of hesperidin and narirutin in commercial orange juice.

## Conclusions

Orange juice is considered to be a health-promoting beverage, especially with pulp. However, in this study, the fibre content was similar with and without pulp. The pulp increased recovery of flavanone during the upper GI digestion, but did not affect degradation of flavanone and production of phenolic acids over 24 h of colonic fermentation, although at 2 h there was a higher level of hesperidin in OJP. The orange juices highly enriched with pulp may have higher phenolic content than pulp-free juices. However, our study highlights that those orange juices with added pulp on sale in UK supermarkets have no greater impact on gut metabolism than juice without pulp. To improve consumer understanding of any potential health benefits of orange juice with pulp, it should be clear how much pulp is added to the juice and if this is enough to affect gut metabolism of hesperidin and narirutin, which should be further studied. More information is needed to enable informed choice for consumers if the health properties of polyphenols are considered to be of importance.

## Electronic supplementary material

ESM 1 (DOCX 69 kb)

## Abbreviations

GAE: Gallic acid equivalents
GI: Gastrointestinal tract
HPLC-PDA: High performance liquid chromatography-photodiode array detector
OJ: Orange juice without pulp
OJP: Orange juice with pulp
