# Supplementary material for: Pulp in Shop-Bought Orange Juice Has Little Effect on Flavonoid Content and Gut Bacterial Flavanone Degradation In Vitro
Source: Plant Foods Hum Nutr. 2019 Jun 21;74(3):383–90. doi: 10.1007/s11130-019-00739-5 (PMC6684804; doi:10.1007/s11130-019-00739-5)
Supplement: Supplementary file 1 — (DOCX 69 kb) [file 11130_2019_739_MOESM1_ESM.docx]

**Pulp in shop bought orange juice had little effect on flavonoid content and gut bacterial flavanone degradation *in vitro***

**Supplemental Material**

Min Hou ^1, 2^, Emilie Combet ^1^, Christine Ann Edwards ^1*^

1. *Human Nutrition, School of Medicine, Dentistry and Nursing, College of Medical, Veterinary & Life Sciences University of Glasgow, Glasgow, UK*
2. *School of Public Health, College of Medicine, Shanghai Jiao Tong University, Shanghai, China*

^*^Corresponding author Professor Christine Edwards

Address: *Human Nutrition, School of Medicine, Dentistry and Nursing, College of Medical, Veterinary & Life Sciences University of Glasgow, New Lister Building, Glasgow Royal Infirmary, 10-16 Alexandra Parade, Glasgow, G31 2ER, Scotland, UK*

Christine.edwards@glasgow.ac.uk

**Chemicals and Methods**

HPLC grade methanol was obtained from Rathburn Chemicals (Walkerburn, Borders, UK). Formic acid was purchased from Riedel-deHaen (Seelze, Germany). Hydrochloric acid, sodium bicarbonate, pepsin (from porcine stomach mucosa), pancreatin (from porcine pancreas), bile acids (porcine), tryptone, anhydrous ethyl acetate, dichloromethane (DCM), N-methyl-N- (trimethylsily) trifluoracetateamide, prydine. CaCl_2_^.^2 H_2_O, MnCl_2_^.^4 H_2_O, NH_4_HCO_3_, NaHCO_3_, Na_2_HPO_4_, KH_2_PO_4_, MgSO_4_^.^7 H_2_O, cysteine hydrochloride, NaOH, Na_2_S ^.^ 9 H_2_O, resazurin and N, o-bis (Trimethylsilyl) trifluoroacetamide (BSTFA) + 10 % trimethylchlorosilane (TMCS) were purchased from Sigma-Aldrich Co Ltd (Poole, Dorset, UK). Ethyl acetate and dichloromethane (DCM) were purchased from Rathburn Chemicals Ltd (Walkerburn, Peeblesshire, UK). 2, 4, 5- trimethoxycinnamic acid was obtained from Fisher Scientific (Loughborough, UK).

Hesperetin-7-O-rutinoside and narigenin-7-O-rutinoside were purchased from Extrasynthese (Genay, France). 4-hydroxyphenylbenzoic acid, 4-hydroxyphenylacetic acid, dihydrofeulic acid were purchased from Sigma-Aldrich Co Ltd (Poole, Dorset, UK). 4-hydroxyphenylpropionic acid and 3,4-dihydroxyphenylpropionic acid were obtained from Alfa Aesar (Karlsruhe, Germany).

**Orange Juices for Analysis**

A selection of OJ and OJP were purchased from local supermarkets in Glasgow, UK between April of 2013 and March of 2014. The juices were labelled 100 % freshly squeezed, not from concentrate, and included Tropicana (Tropicana UK Ltd Leicester UK), Sainsbury’s (Sainsbury’s Supermarkets Ltd London UK), Tesco (Dundee UK), Innocent (London UK), Waitrose (Bracknell UK) and Morrison’s (Bradford UK) orange juices. These brands provide commercial OJ and OJP.

***In vitro* Gastrointestinal Digestion Model**

Tropicana juices were selected as a representative brand for investigating *in vitro* digestion and fermentation of orange juice in the GI, because of there large consumption in UK. The volumes of orange juices used in the models representing each part of the GI were determined to reflect the ingestion of 250 ml juice [1].

The model of the upper GI digestion was adapted from Gil-Izquierdo [2]. For the gastric digestion, 40 ml orange juice was adjusted to pH 2 by adding HCl (5 M), and pepsin (0.32 % w/v) to cap-sealed bottles, and then incubated at 37 °C in a shaking water bath at 100 stokes/min for 1 h. Aliquots (1.5 mL) were taken before and after the gastric digestion and stored at -80 °C until analysis. After the gastric phase, the orange juice digesta was neutralized with NaHCO_3_ before addition of pancreatin (4 g/l) and bile extract (25 g/l) in 0.1 M NaHCO_3_. Incubations were performed at 37 °C for 6 h in a shaking water bath at 100 stokes/min. Aliquots (1.5 mL) were taken at 2 h, 4 h and 6 h during the pancreatin / bile acids digestion and stored at -80 °C until analysis. The GI digestion was conducted in triplicate.

After the small intestinal digestion model, the digesta were dialysed using cellulose dialysis tubing (molecular weight cut-off 0.1-0.5 kDa, Sigma) into 5 L of distilled water to remove sugars and dextrins, fatty acids and peptides normally be absorbed in the small intestine (but not flavonoids (hesperidin: MW 610 and narirutin: MW 580). Dialysed samples were then freeze-dried (Edwards Micro Modulyo, Crawley, UK) and the residue used as the substrate in the fermentation model. The dialysis membrane was not used for evaluation of stability/ release of hesperidin and narirutin from orange juice during gastrointestinal digestion [3].

***In vitro* fermentation with human colonic bacteria**

Faecal samples were obtained from six healthy Caucasian volunteers (four women and two men, 23 ± 2.6 years, 67 ± 12.5 kg, BMI 22.5 ± 2.3) who were non-smokers, had a normal diet, no digestive diseases, no food allergies, and did not receive antibiotics for 6 months prior to recruitment. Volunteers followed a low-polyphenol diet for 2 days to avoid fruits, vegetables, tea, coffee, wine and high fibre foods before providing a faecal sample.

Fresh faeces were homogenized with sodium phosphate buffer (pH 7) to obtain 32 % faecal slurry. Faecal slurries were then strained through nylon mesh to remove particles. Strained slurry (5 ml) was added to fermentation medium. The fermentation medium contained buffer, macro and micro minerals, tryptone, reazurin (42 ml) and reducing solution (2 ml; cysteine HCL and Na_2_S) [4]. The fermentation bottles were sealed air-tight with polyethylene caps, and purged with OFN until anaerobic conditions were obtained, before incubation for 24 h at 37 °C in a shaking water bath at 60 stokes/min. All fermentations were conducted in duplicate. Aliquots were collected at 0, 2, 4, 6, 24 h, and stored at - 80 °C until analysis.

**Analysis of Fibre in OJ and OJP**

The fibre content of each orange juice was analysed by using AOAC method [5] by the commercial food testing facility [6] at the School of Health and Life Science, Glasgow Caledonian University, Glasgow UK.

**Analysis of Total Flavonoids**

Total flavonoids were determined in orange juices by spectrophotometric method based on production of complex compounds of flavonoids with aluminium chloride [7], using a microplate method, and a Multiskan spectrum plate reader (Thermo Fisher Scientific Inc., Stafford, UK).The quercetin standard curve ranged from 50 to 200 μg ml^-1^.

**Analysis of Total Phenols**

The amount of total phenols in orange juices and samples from *in vitro* GI digestion was determined by the Folin-Ciocalteau method [8], using a microplate method, and a multiskan spectrum plate reader (Thermo Fisher Scientific Inc., Stafford, UK) in triplicate. Aqueous solutions of gallic acid concentration ranging from 50 to 800 µg ml^-1^ were used as standard curve.

**Analysis of Antioxidant Capacity**

Antioxidant capacity was determined in orange juices and samples from *in vitro* GI digestion using the FRAP assay in a redox-linked colorimetric reaction [9], using a microplate method, and a multiskan spectrum plate reader (Thermo Fisher Scientific Inc., Stafford, UK) in triplicate. Aqueous solutions of known Fe^2+^ (FeSO_4_∙7H_2_O) concentration ranging from 0.2 to 1 mmol/l were used as standard curve.

**Extraction of OJ and OJP**

Orange juices (1 mL) were mixed with 1 ml of methanol for 10 min by using an Ultraturrax homogenizer (Fisher Scientific, UK). The mixture was centrifuged at 4500 g for 10 min at 4 °C. The pellet was extracted with 0.5 mL of methanol in duplicate. The extracts were evaporated centrifugally under vacuum at room temperature using a Thermo Speedvac Savant concentrator (Thermo Fisher Scientific, Stafford, UK). The residue was re-dissolved with 25 μl of methanol and made up to 1 mL with 0.1 % formic acid for analysis.

**Extraction of the gastric digesta and the fermented samples**

Samples were thawed and homogenized with a Disruptor Genie (Scientific Industries, UK). Sample (1 mL) and methanol (1 mL) containing 1 % formic acid were mixed in a glass tube for 10 min at room temperature using an orbital shaker (Vibrax VXR Z04063 Aldrich, UK). The mixtures were then centrifuged at 13,000 rpm at 4 °C for 10 min. The extraction with methanol from the pellet was repeated in duplicate. Supernatants were combined and centrifugally evaporated under vacuum at room temperature using a Thermo Speedvac concentrator (Thermo Fisher Scientific, Stafford, UK). Residues were then re-dissolved with 25 μl of methanol and 475 μl of 0.1% formic acid for analysis by high performance liquid chromatography-photodiode array detector (HPLC-PDA).

**Analysis of hesperidin, hesperein, narirutin and naringenin in orange juice, digesta and fermented samples by HPLC-PDA**

Analysis of hesperidin, hesperetin, narirutin and naringenin was performed as described by Pereira-Caro et al [10]. Samples were injected onto a Thermo Surveyor HPLC system comprising an HPLC pump, PDA detector scanning from 250 to 700 nm, and an autosampler cooled to 4 °C (Thermo Finnigan, San Jose, CA, USA). Separation was carried out using a 250 × 4.6 mm i.d. 4 μm Synergi Max-RP column (Phenomenex, Macclesfield, UK) and eluted from 5 to 50 % methanol in 0.1% formic acid within 60 min at a flow rate of 1 ml/min and maintained at 40 °C. Hesperidin, narirutin, hesperetin and narigenin were identified based on retention times of individual authentic standards. Peak area versus concentration was obtained by linear regression analysis of individual authentic standards concentration ranging from 0.1 ng µl^-1^ to 300 ng µl^-1^ at 290 nm.

**Extraction and Derivatization of Phenolic Acids in** **fermented samples**

Phenolic acid extraction, derivatisation and analysis were performed as described by Combet et al [11]. Samples and calibration standards (0.5 ml) were mixed with 30 µl of 2, 4, 5-trimethoxycinnamic acid (TMCA) as an internal standard and 60 µl of aqueous 1M HCl. Mixtures were vortexed and then placed at 4 °C for 10 min. Anhydrous ethyl acetate (1.5 mL) was added and vortexed for 30 s, and then centrifuged at 2700 g for 10 min at 4 °C. The upper organic layer was transferred to an amber glass vial in a 37 °C aluminium block and dried under a gentle flow of nitrogen gas. The residue solutions were extracted as above, and upper layers were transferred to the same amber vial and dried under a gentle flow of nitrogen. Dichloromethane (DCM) (200 µl) was added to rinse vial walls and dried under a slight stream of nitrogen. Derivatization reagent (50 µl) [N, o-Bis (Trimethylsilyl) trifluoroacetamide (BSTFA) + 10 % trimethylchlorosilane (TMCS)] was added to the amber vials. The headspace was flushed with a gentle flow of nitrogen before sealing. Samples were vortexed each 30 min at 70 °C for 4 h. Anhydrous hexane (350 µl) was added after derivatisation for analysis.

**Analysis of phenolic acids in fermented samples by GC-MS**

Derivatised phenolic acids were analysed using a Trace DSQ single quadruple GC-MS, equipped with an AI 300 autosampler (Thermo Finnigan Ltd, Hempstead, Hertfordshire, UK). Samples were injected in the split mode with a 25:1 ratio. The injector temperature was maintained at 220 °C. The mass spectrometer was used in the positive ionization mode with the ion source and transfer line set at 180 °C and 310 °C, respectively. Separations were carried out on a fused silica capillary column (30 m × 0.25 mm i.d.) coated with cross-linked 5 % phenylmethylsiloxane (film thickness 0.25 µm) (Phenomenex, Macclesfield, Cheshire, UK). Helium was the carrier gas with a flow rate of 1.2 ml min^-1^. The column temperature was initially set at 40 °C and raised to 160 °C at 20 °C min^-1^, 200 °C at 1.5 °C min^-1^ and 250 °C at 10 °C min^-1^ to a final temperature of 300 °C at 40 °C min^-1^, held for 5 min. Data acquisition was performed in full scan mode (m/z 50 - 470) with ionization energy of 70 eV, and analysis was carried out by using Xcalibur software version 2.0 (Thermo Fisher Scientific UK Hempstead, Hertfordshire, UK). Phenolic acids were identified based on the mass spectra and retention time of authentic standards analysed under identical conditions. When standards were not commercially available, identification was achieved by comparison with data from the integrated NIST mass spectral library 2008 (Scientific Instruments Services Inc., Ringoes, NJ, USA), with a confidence of 70 % or above. Calibration curves of the ratio between the target ion (m/z) of the standard compound of interest and the target ion of the internal standard (m/z 279) were computed, with concentrations ranging from 3- 40 mg ml-1 (R^2^ > 0.95).

**References**

1. Mullen W, Archeveque MA, Edwards CA, Matsumoto H, Crozier A (2008) Bioavailability and metabolism of orange juice flavanones in humans: impact of a full-fat yogurt. J Agric Food Chem 56 (23):11157-11164.

2. Gil-Izquierdo A, Gil MI, Tomás-Barberán FA, Ferreres F (2003) Influence of industrial processing on orange juice flavanone solubility and transformation to chalcones under gastrointestinal conditions. J Agric Food Chem 51 (10):3024-3028

3. Bermúdez-Soto M-J, Tomás-Barberán F-A, Garcia-Conesa M-T (2007) Stability of polyphenols in chokeberry (*Aronia melanocarpa*) subjected to *in vitro* gastric and pancreatic digestion. Food Chem 102 (3):865-874

4. Jaganath IB, Mullen W, Lean MEJ, Edwards CA, Crozier A (2009) In vitro catabolism of rutin by human fecal bacteria and the antioxidant capacity of its catabolites. Free Radic Biol Med 47 (8):1180-1189.

5. McCleary BV, DeVries JW, Rader JI, Cohen G, Prosky L, Mugford DC, Okuma K (2012) Determination of insoluble, soluble, and total dietary fiber (CODEX definition) by enzymatic-gravimetric method and liquid chromatography: collaborative study. Journal of AOAC International 95 (3):824-844

6. Mayer R, Stecher G, Wuerzner R, Silva RC, Sultana T, Trojer L, Feuerstein I, Krieg C, Abel G, Popp M (2008) Proanthocyanidins: target compounds as antibacterial agents. J Agric Food Chem 56 (16):6959-6966

7. Chang C-C, Yang M-H, Wen H-M, Chern J-C (2002) Estimation of total flavonoid content in propolis by two complementary colorimetric methods. Journal of food and drug analysis 10 (3)

8. Singleton V, Rossi JA (1965) Colorimetry of total phenolics with phosphomolybdic-phosphotungstic acid reagents. Am J Enol Viticul 16 (3):144-158

9. Benzie IF, Strain J (1996) The ferric reducing ability of plasma (FRAP) as a measure of “antioxidant power”: the FRAP assay. Anal Biochem 239 (1):70-76

10. Pereira-Caro G, Borges G, Van Der Hooft J, Clifford MN, Del Rio D, Lean ME, Roberts SA, Kellerhals MB, Crozier A (2014) Orange juice (poly) phenols are highly bioavailable in humans. The Am J Clin Nutr 100 (5):1378-1384

11. Combet E, Lean ME, Boyle JG, Crozier A, Davidson DF (2011) Dietary flavonols contribute to false-positive elevation of homovanillic acid, a marker of catecholamine-secreting tumors. Clin Chim Acta 412 (1):165-169


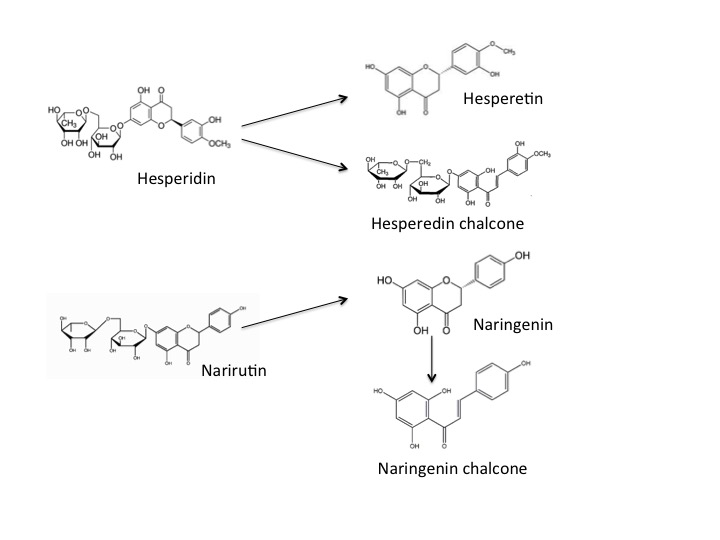


Fig S1 Structure of glycosides, aglycones and chalcone of hesperetin and naringenin
